# Supplementary material for: Combining 16S Sequencing and qPCR Quantification Reveals Staphylococcus aureus Driven Bacterial Overgrowth in the Skin of Severe Atopic Dermatitis Patients
Source: Biomolecules. 2023 Jun 23;13(7):1030. doi: 10.3390/biom13071030 (PMC10377005; doi:10.3390/biom13071030)

Table S1. **Demographic data for the longitudinal dataset (A) and cross-sectional dataset (B).**

(A)

Longitudinal dataset	AD Status		All	Fisher or MW test (p-value)
	Healthy	AD		
**Gender**				
**Female (n)**	4	4	8	ns
**Male (n)**	2	2	4	
**Age, years (Median, range)**	40.1 (28-68)	43.2 (22-66)	42.9 (22-68)	ns
**N**	6	6	**12**	--

* exclusion of 1 individual in the HE due to atopic condition.

(B)

Cross-sectional dataset	Healthy	AD All	AD by Severity			Fisher or KW test (p-value)
			Mild	Moderate	Severe	
**Gender**						
**Female (n)**	12	95	35	36	24	ns
**Male (n)**	8	40	10	15	15	
**Age, years (median, range)**	40 (20-69)	29 (0-80)	27 (0-76)	37 (0-80)	31 (4-80)	ns
**N**	**20**	**135**	45	51	39	--

ns = not statistically significant

Figure S1. **Correlation between the relative *S. aureus* abundance observed via NGS or qPCR in the longitudinal dataset (A) and cross-sectional dataset (B).** UD=undetectable.

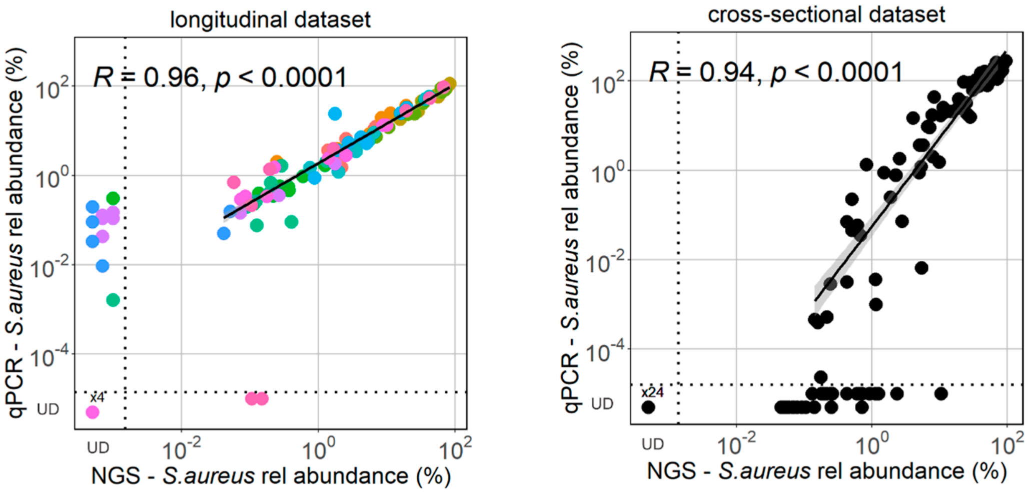

(A) **(B)**

Figure S2: Kinetics of *S. aureus* absolute and relative abundance for all 6 AD patients in the longitudinal dataset.

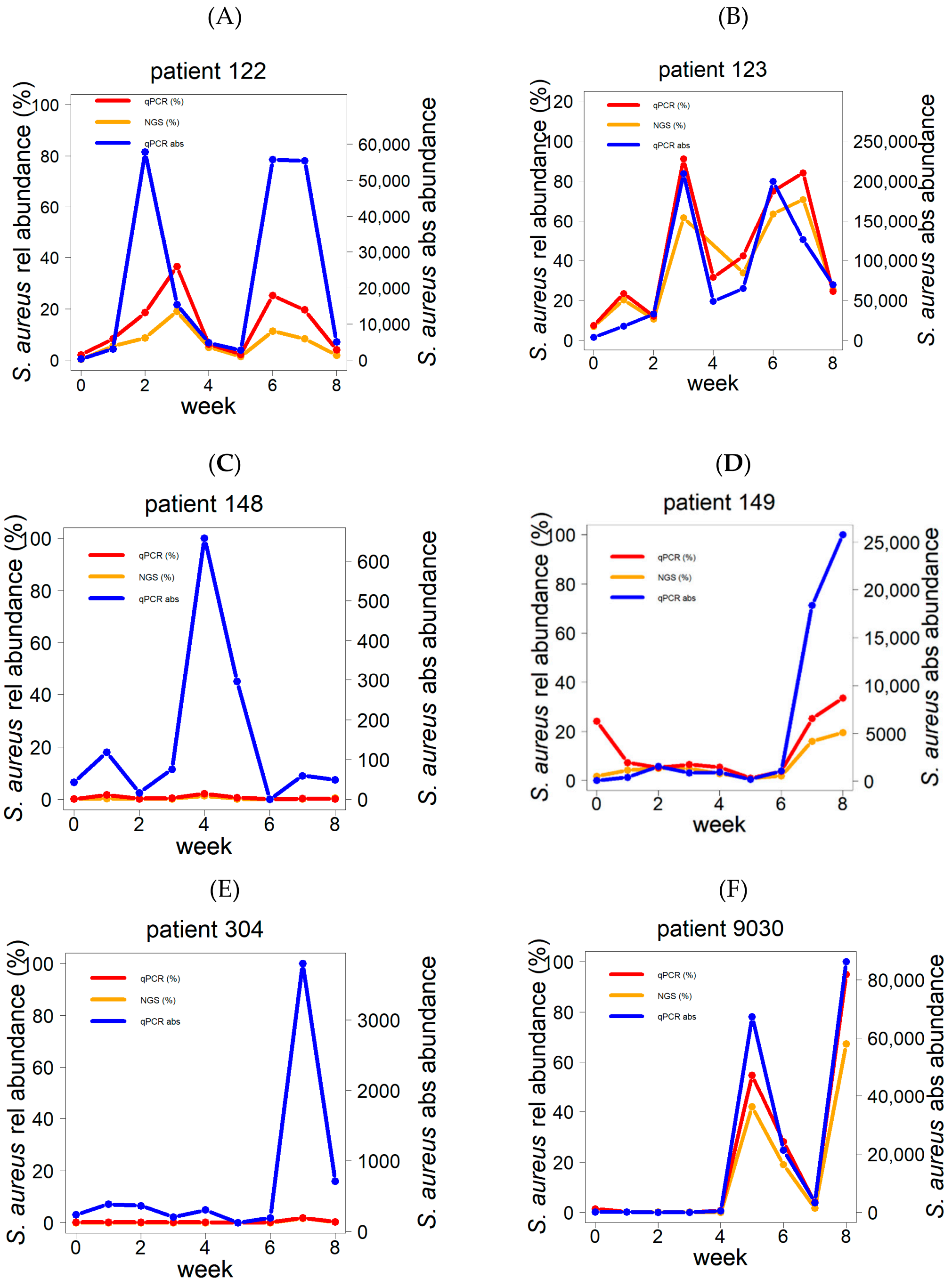

Figure S3: *S. aureus* absolute (A) and relative (B) abundance and 16S copy number (C) as function of age and AD severity in the cross-sectional dataset.

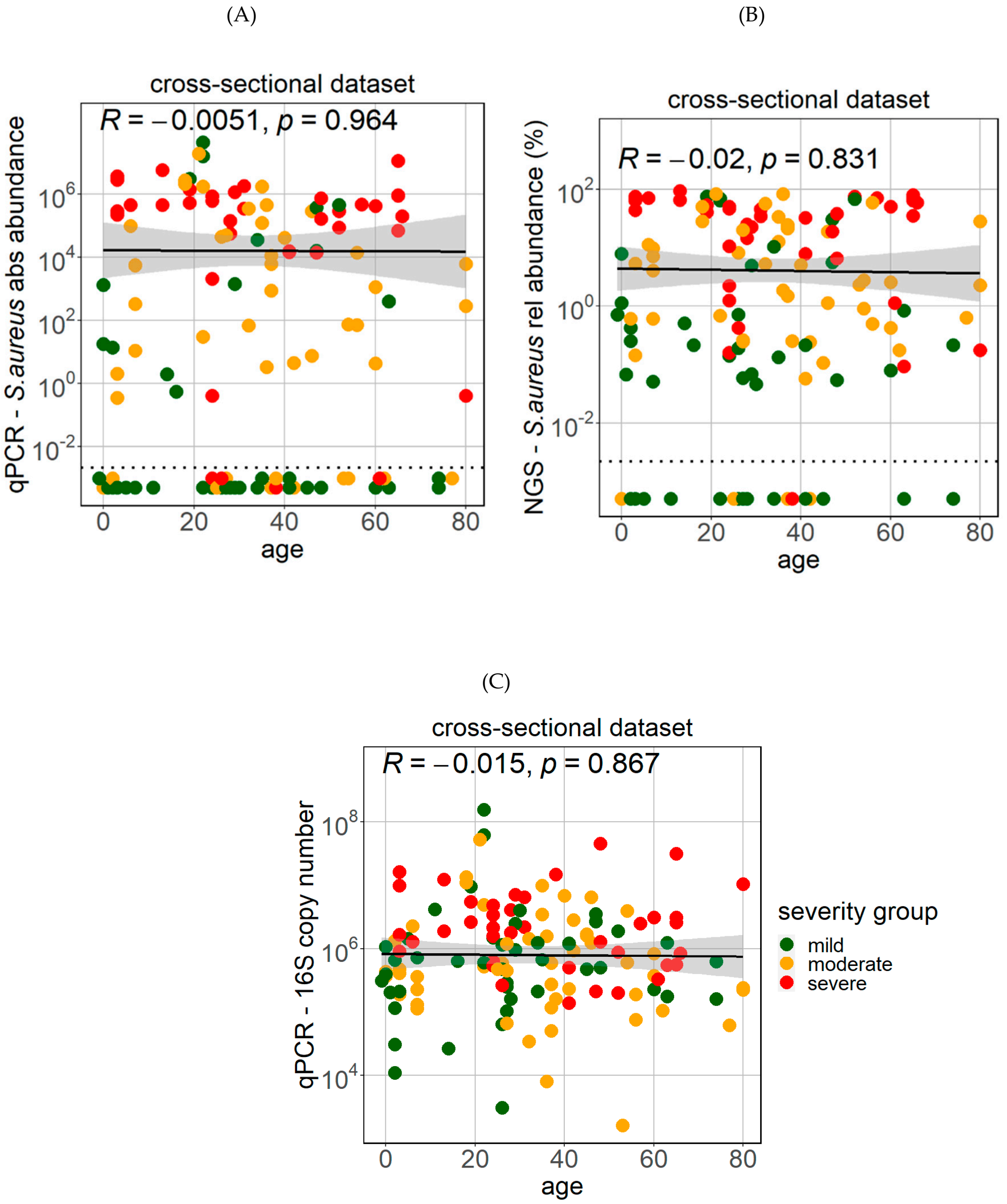

Supplement: Supplementary file 1 [file biomolecules-13-01030-s001.zip › biomolecules-2392848-supplementary.pdf]
